# Absence of Strong Genetic Linkage Disequilibrium between Single Nucleotide Polymorphisms (SNPs) in the Prion Protein Gene (*PRNP*) and the Prion-Like Protein Gene (*PRND*) in the Horse, a Prion-Resistant Species

**DOI:** 10.3390/genes11050518

**Published:** 2020-05-07

**Authors:** Sae-Young Won, Yong-Chan Kim, Kyoungtag Do, Byung-Hoon Jeong

**Affiliations:** 1 Korea Zoonosis Research Institute, Jeonbuk National University, Iksan, Jeonbuk 54531, Korea; gkfh32@jbnu.ac.kr (S.-Y.W.); kych@jbnu.ac.kr (Y.-C.K.); 2 Department of Bioactive Material Sciences, Jeonbuk National University, Jeonju, Jeonbuk 54896, Korea; 3 Lab of Equine Science, Department of Animal Biotechnology, Faculty of Biotechnology, Jeju National University, Jeju 63243, Korea; challengekt@gmail.com

**Keywords:** prion, polymorphisms, single nucleotide polymorphisms, prion-like protein gene, Doppel, *PRND*, *PRNP*

## Abstract

Prion disease is a fatal neurodegenerative disorder caused by a deleterious prion protein (PrP^Sc^). However, prion disease has not been reported in horses during outbreaks of transmissible spongiform encephalopathies (TSEs) in various animals in the UK. In previous studies, single nucleotide polymorphisms (SNPs) in the prion protein gene (*PRNP*) have been significantly associated with susceptibility to prion disease, and strong linkage disequilibrium (LD) between *PRNP* and prion-like protein gene (*PRND*) SNPs has been identified in prion disease-susceptible species. On the other hand, weak LD values have been reported in dogs, a prion disease-resistant species. In this study, we investigated SNPs in the *PRND* gene and measured the LD values between the *PRNP* and *PRND* SNPs and the impact of a nonsynonymous SNP found in the horse *PRND* gene. To identify SNPs in the *PRND* gene, we performed direct sequencing of the *PRND* gene. In addition, to assess whether the weak LD value between the *PRNP* and *PRND* SNPs is a characteristic of prion disease-resistant animals, we measured the LD value between the *PRNP* and *PRND* SNPs using D’ and *r*^2^ values. Furthermore, we evaluated the impact of a nonsynonymous SNP in the Doppel protein with PolyPhen-2, PROVEAN, and PANTHER. We observed two novel SNPs, c.331G > A (A111T) and c.411G > C. The genotype and allele frequencies of the c.331G > A (A111T) and c.411G > C SNPs were significantly different between Jeju, Halla, and Thoroughbred horses. In addition, we found a total of three haplotypes: GG, AG, and GC. The GG haplotype was the most frequently observed in Jeju and Halla horses. Furthermore, the impact of A111T on the Doppel protein was predicted to be benign by PolyPhen-2, PROVEAN, and PANTHER. Interestingly, a weak LD value between the *PRNP* and *PRND* SNPs was found in the horse, a prion disease-resistant animal. To the best of our knowledge, these results suggest that a weak LD value could be one feature of prion disease-resistant animals.

## 1. Introduction

Prion diseases are fatal neurodegenerative disorders that produce an abnormal deleterious prion protein (PrP^Sc^) from a normal prion protein (PrP^C^) [1,2,3,4]. The prion protein (PrP) is encoded by the prion protein gene (*PRNP*), and polymorphisms in the *PRNP* gene affect the susceptibility of prion diseases in humans and ruminants [5,6,7,8,9,10,11]. In humans, susceptibility to sporadic and variant Creutzfeldt–Jakob disease (CJD) is related to *PRNP* codon 129 methionine homozygosity and/or 219 glutamic acid homozygosity [12,13]. In addition, the vulnerability of sheep scrapie is associated with valine at codon 136, arginine at codon 154 and glutamine at codon 171 (VRQ) and Alanine at codon 136, Arginine at codon 154 and glutamine at codon 171 (ARQ) haplotypes at *PRNP* codons 136, 154, and 171 [14,15,16]. Over 40 polymorphisms in the *PRNP* gene have been reported in goats and sheep. Among them, 7 polymorphisms, namely, 142, 143, 146, 154, 171, 211, and 222, are related to prion vulnerability [17,18,19]. In contrast, recent studies have reported that prion disease-resistant species, such as horses in perissodactyls and dogs in carnivores, have distinct genetic features of the *PRNP* gene [20,21]. Compared with prion disease-susceptible species, dogs have a unique dog-specific amino acid, aspartic acid (D) located at *PRNP* codon 163, which affects prion resistance [22]. In addition, horses have a distinct structure of the PrP, *β*2-*α*2 loop, which contributes to the structural stability of horse PrP [23]. In addition, replacing the dog-specific amino acid D159 and horse-specific amino acid S167 showed toxicity in a transgenic model of dog and horse PrP, respectively [24]. Furthermore, a recent study found that four salt bridges in horse PrP also contribute to its structural stability [25]. However, spontaneous prion aggregation has been observed in mice with a specific amino acid of the horse [26]. Thus, the hypothesis, which can explain prion disease with horse PrP alone, is complicated. Therefore, we investigated the prion-like protein gene (*PRND*), which is related to the *PRNP* gene.

Recent studies have reported a strong genetic linkage of single nucleotide polymorphisms (SNPs) between the *PRNP* gene and the *PRND* gene in prion disease-susceptible species. Strong linkage disequilibrium (LD) between *PRNP* codons 136, 154, and 171 and *PRND* codon 26 was first reported in Portuguese sheep. The alanine at codon 136, arginine at codon 154 and arginine at codon 171 (ARR) haplotype of the *PRNP* gene is associated with resistance to prion infection and significantly linked with the G allele at *PRND* codon 26. In contrast, the ARQ haplotype is associated with susceptibility to prion infection and linked with the A allele at codon 26 of the *PRND* gene [27]. In addition, in prion disease-susceptible animals, goats also have strong LD between codon 143 of the *PRNP* gene and codons 28, 151, and 385 of the *PRND* gene and between codon 102 of the *PRNP* gene and codon 99 of the *PRND* gene, respectively [28]. However, in prion disease-resistant species such as dogs, all SNPs in the *PRND* gene show weak LD with all SNPs in the *PRNP* gene (*r*^2^ value: below 0.3) [29]. However, it is elusive whether the weak LD value between the *PRNP* gene and the *PRND* gene in dogs is characteristic of prion disease-resistant species.

In our previous study, we did not discover SNPs in the horse *PRND* gene in the Thoroughbred breed [30]. Because the Thoroughbred breed is known to strictly regulate mating, the genetic distribution of the *PRND* gene may be biased. Thus, we investigated outbred horses, Jeju and Halla horses, to validate the absence of polymorphisms in the open reading frame (ORF) of the *PRND* gene in horses. The Jeju horse is a Korean native horse that may have originated from Mongolian or independent lineage [31]. Halla horses are a crossbreed of the Jeju and Thoroughbred horses with the purpose of horse racing [32]. 

In this study, we investigated SNPs using direct sequencing of the horse *PRND* gene in Jeju and Halla horses. We also analyzed the genotype, allele, and haplotype frequencies of horse *PRND* SNPs and compared their distributions between Thoroughbred, Jeju, and Halla horses. In addition, we evaluated the influence of the identified nonsynonymous SNPs in the horse *PRND* gene using PolyPhen-2, PROVEAN, and PANTHER [33,34,35,36]. Furthermore, we measured the LD value between the *PRNP* and *PRND* SNPs in horses to assess whether strong genetic linkage is a characteristic of prion disease-resistant animals.

## 2. Materials and Methods

### 2.1. Ethical Statement

The hair samples of 148 Jeju and 100 Halla horses were collected from Jeju Island. All experimental procedures were approved by the Institute of Animal Care and Use Committee of Jeonbuk National University (JBNU 2016-65). All experiments using horses were carried out following the Korea Experimental Animal Protection Act.

### 2.2. Genetic Analysis

The genomic DNA of Halla and Jeju horses was isolated from 10 hair bulbs using a HiYield™ Genomic DNA Mini Kit (Real Biotech Corporation, Banqiao city, Taiwan) according to the manufacturer’s instructions. Polymerase chain reaction (PCR) on the horse *PRND* coding region located at exon 3 was performed with sense and antisense primers as follows: horse *PRND* forward 5′-GCCCGTTGCAGCTTCTTATCT-3′ and horse *PRND* reverse 5′-GCTGGAGGAGAGAAGTGGGAT-3′. PCR was performed in a final volume of 25 µL, including 1 µL of genomic DNA, 10 pmol of each primer, 2.5 µL of 10X *Taq* DNA polymerase buffer, 0.5 µL of a 0.2 µM dNTP mixture, 2.5 µL of 5X band helper, and 0.2 µL *Taq* DNA polymerase. The PCR conditions were as follows: denaturing at 95 °C for 2 min, 33 cycles of 95 °C for 20 s, 61 °C for 40 s, 72 °C for 1 min 30 s, and 1 cycle 72 °C for 5 min. The purification of PCR products was performed using a FavorPrep GEL/PCR Purification Mini Kit (FAVORGEN, Pingtung city, Taiwan). The PCR products were directly sequenced using an ABI 3730 sequencer (ABI, Foster City, CA, USA), and sequencing results were analyzed using Finch TV software (Geospiza, Inc., Seattle, WA, USA).

### 2.3. Statistical Analysis

The genotype, allele, and haplotype frequencies of the horse *PRND* gene were compared between 3 horse breeds by the chi-square test using SAS 9.4 software. In addition, we examined LD calculated by Lewontin’s D’ (|D’|), and pairwise linkage disequilibrium (*r*^2^) was examined using Haploview version 4.2 (Broad Institute, Cambridge, MA, USA). The Hardy–Weinberg equilibrium (HWE) test and haplotype analysis were carried out using Haploview version 4.2 (Broad Institute, Cambridge, MA, USA).

### 2.4. Analysis of Genetic Linkage between SNPs in the PRNP and PRND Genes

We performed LD analysis between *PRNP* and *PRND* SNPs in 135 Jeju and 72 Halla horses. In addition, we examined LD calculated by Lewontin’s D’ (|D’|), and pairwise linkage disequilibrium (*r*^2^) was examined using Haploview version 4.2 (Broad Institute, Cambridge, MA, USA).

### 2.5. Measurement of Protein Functional Alterations Induced by Nonsynonymous SNPs

We predicted the damaging effect of amino acid substitution induced by nonsynonymous SNPs using the PolyPhen-2 v.2.2.2, PROVEAN v.1.1.3, and PANTHER 15.0 programs. PolyPhen-2 evaluates the effect of amino acid changes from an independent count (PSIC), represented as a score ranging from 0 (tolerated) to 1 (deleterious). The prediction range of 0.0 to 0.15 indicates ‘benign’, 0.15 to 1.0 indicates “possibly damaging”, and 0.85 to 1.0 indicates “damaging”. PROVEAN estimates nonsynonymous or indel variants; a score below −2.5 is considered “deleterious”, and a score above −2.5 is considered “neutral”. PANTHER classifies amino acids into groups with similar molecular biology. In PANTHER, a score below −3 indicates “deleterious” and a score above −3 indicates “neutral”.

### 2.6. Physical Map Distance between the PRNP and PRND Genes among Several Species

Physical map distances between the *PRNP* and *PRND* gene sequences were investigated in humans (*Homo sapiens*, *PRNP* gene [Gene ID: 5621], *PRND* gene [Gene ID: 23627]), sheep (*Ovis aries*, *PRNP* gene [Gene ID: 493887], *PRND* gene [Gene ID: 443194]), goats (Capra hircus, *PRNP* gene [Gene ID: 102169975], *PRND* gene [Gene ID: 102170246]), dogs (*Canis lupus familiaris*, *PRNP* gene [Gene ID: 485783], *PRND* gene [Gene ID: 485782]), and horses (*Equus caballus*, *PRNP* gene [Gene ID: 100065904], *PRND* gene [Gene ID: 100048937]). We obtained sequence information from GenBank at the National Center for Biotechnology Information (NCBI).

## 3. Results

### 3.1. Investigation of Polymorphisms in the Horse PRND Gene in 140 Jeju and 80 Halla Horses

**Figure 1:**
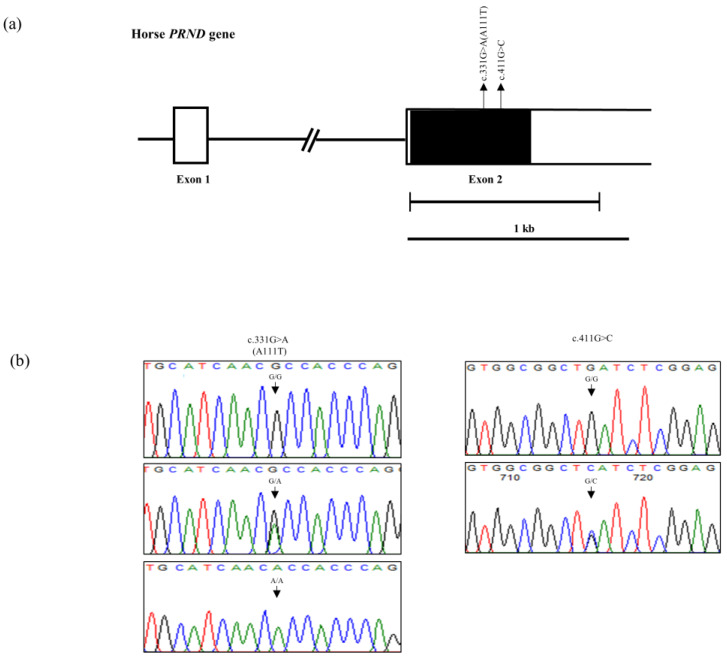
Gene map and polymorphisms identified in the horse prion-like protein (*PRND*) gene on chromosome 22. (**a**) The open reading frame (ORF) is indicated by a shaded block, and the 5′ and 3′ untranslated regions (UTRs) are represented by white blocks. Arrows indicate the novel polymorphisms found in this study. (**b**) Electropherograms showing the two novel single nucleotide polymorphisms (SNPs) identified in this study. left panels; c.331G > A (A111T), right panels; c.411G > C.

The horse *PRND* gene consists of two exons, and exon 2 contains the ORF. We investigated polymorphisms in exon 2 of the *PRND* gene in two horse breeds, Jeju and Halla. The *PRND* gene sequences of Halla and Jeju horses are identical to that of *Equus caballus* registered in GenBank (Gene ID: 100048937). We discovered a total of two novel SNPs, c.331G > A and c.411G > C. c.331G > A (A111T) is a nonsynonymous SNP, and c.411G > C is a synonymous SNP (Figure 1a,b). The genotype and allele frequencies of the *PRND* SNPs are described in Table 1 and Table 2. Both SNPs were in HWE.

**Figure 2:**
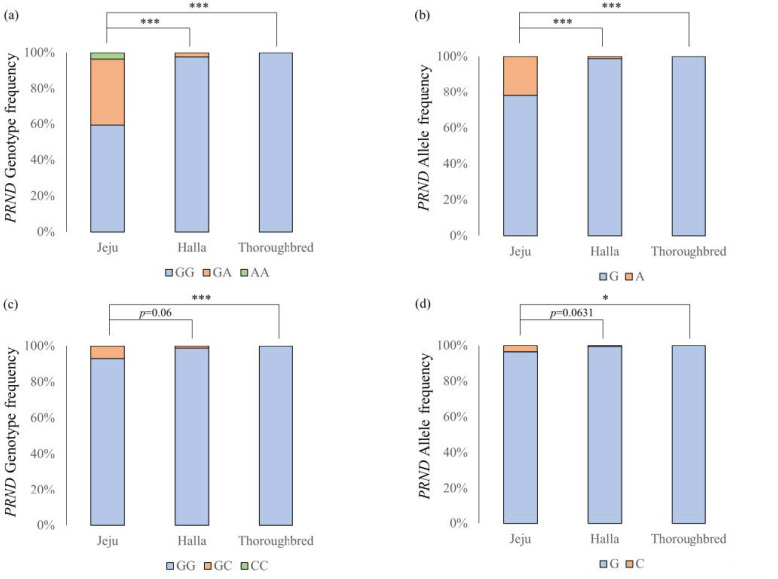
Comparisons of genotype and allele frequencies of horse prion-like protein (*PRND*) gene SNPs between Jeju, Halla, and Thoroughbred horses. (**a**) Comparison of the genotype frequencies of the *PRND* c.331G > A (A111T) polymorphism between Jeju, Halla, and Thoroughbred horses. (**b**) Comparison of the allele frequencies of the *PRND* c.331G > A polymorphism between Jeju, Halla, and Thoroughbred horses. (**c**) Comparison of the genotype frequencies of the *PRND* c.411G > C polymorphism between Jeju, Halla, and Thoroughbred horses. (**d**) Comparison of the allele frequencies of the *PRND* c.411G > C polymorphism between Jeju, Halla, and Thoroughbred horses. Statistically significant differences are indicated below. *: *p* value < 0.05, ***: *p* value < 0.001.

Next, we compared the genotype and allele frequencies of the c.331G > A (A111T) and c.411G > C SNPs in the horse *PRND* gene in Jeju, Halla, and Thoroughbred horses. Regarding the c.331G > A (A111T) SNP, the genotype and allele frequencies in the Jeju horse were significantly different from those in the Halla (*p* < 0.0001) and Thoroughbred (*p* < 0.0001) horses. However, regarding the c.411G > C SNP, the genotype distribution in the Jeju horse showed no significant difference with that in the Halla horse (*p* = 0.06) but showed a significant difference with that in the Thoroughbred horse (*p* < 0.0001). In addition, the allele frequency in the Jeju horse showed no significant difference with that in the Halla horse (*p* = 0.0631) but showed a significant difference with that in the Thoroughbred horse (*p* = 0.0022) (Figure 2, Table 1 and Table 2).

We performed a haplotype analysis of the two *PRND* SNPs in Jeju and Halla horses. A total of three haplotypes, GG, AG, and GC, were observed in these two horses. The GG haplotype was the most frequently observed in the Jeju (74.3%) and Halla (98.1%) horses. The haplotype frequencies between Jeju and Halla horses were significantly different (*p* < 0.0001) (Table 3). We also estimated the biological impact of the nonsynonymous SNP c.331G > A (A111T) using PolyPhen-2, PROVEAN, and PANTHER. A111T was predicted to be “benign”, with a score of 0.006 by PolyPhen-2, “neutral”, with a score of −0.969 by PROVEAN, and “probably benign”, with a score of 176 by PANTHER (Table 4).

Next, we measured LD of the two horse *PRND* SNPs by (|D’|) and *r*^2^ values. Both SNPs were strongly linked, with a D’ value of 1.0 according to the (|D’|) value in Jeju and Halla horses. However, the *r*^2^ value showed weak LD, with values below 0.02 (Table 5).

### 3.2. Analysis of Genetic Linkage between SNPs in the PRNP and PRND Genes

**Figure 3:**
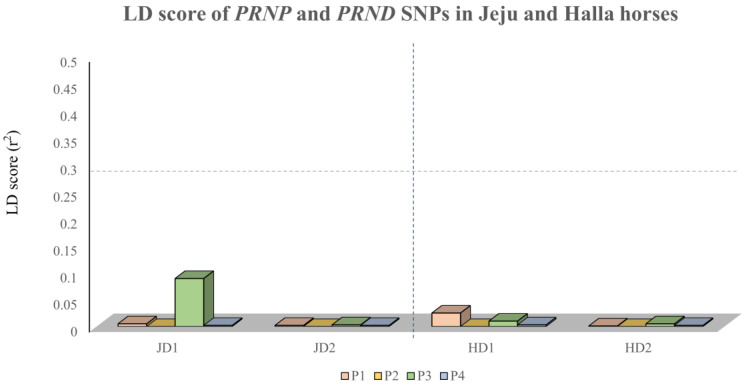
Measurement of linkage disequilibrium (LD) values between *PRNP* and *PRND* SNPs in Jeju and Halla horses. JD1 and HD1 indicate horse *PRND* c.331G > A (codon 111), and JD2 and HD2 indicate horse *PRND* c.411G > C (codon 137). P1 ~ P4 indicate horse *PRNP* SNPs as follows: P1, c.-3A > G; P2, c.301T > A (codon 101); P3, c.525C > A (codon 175); and P4, c.570G > A (codon 190).

To examine whether horse *PRND* SNPs have genetic linkage with the *PRNP* gene, we measured the LD values between SNPs in the *PRNP* and *PRND* genes. All *PRND* SNPs showed weak LD with the *PRNP* SNPs (*r*^2^ value: below 0.1) (Figure 3). The detailed *r*^2^ values are described in Table 6.

### 3.3. Measurement of the Physical Map Distance between the PRNP and PRND Genes among Several Species

**Figure 4:**
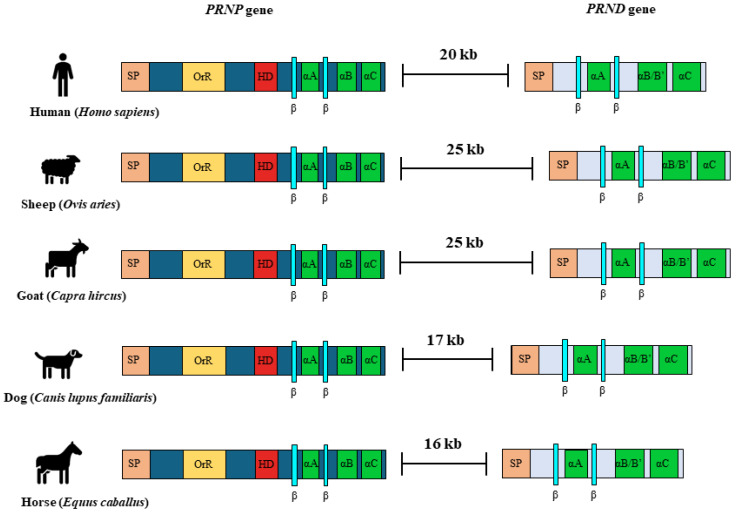
Physical map distance between the *PRNP* and *PRND* genes among several species. Physical map distances between the *PRNP* and *PRND* genes among several species were visualized in prion disease-resistant species, including dogs (*Canis lupus familiaris*) and horses (*Equus caballus*), and prion disease-susceptible species, including humans (*Homo sapiens*), sheep (*Ovis aries*), and goats (*Capra hircus*). SP: Signal peptide; OrR: Octapeptide repeat; HD: Hydrophobic region.

We measured the physical map distance between the *PRNP* and *PRND* genes in prion disease-susceptible species, including humans, sheep, and goats, and prion disease-resistant species, including dogs and horses. Notably, prion disease-resistant species, including dogs (17 kb) and horses (16 kb), showed relatively short physical map distances between the *PRNP* and *PRND* genes compared to those of prion disease-susceptible animals, including humans (20 kb), sheep (25 kb), and goats (25 kb) (Figure 4).

### 3.4. Genetic Linkage between PRNP and PRND Genes among Several Species

Lastly, we summarized the genetic linkage between *PRNP* and *PRND* genes in prion disease-susceptible species, including humans, sheep, and goats, and prion disease-resistant species, including dogs and horses. Notably, prion disease-susceptible animals showed strong genetic linkage between the *PRNP* and *PRND* genes compared to prion disease-resistant species (Table 7).

## 4. Discussion

A previous study surveyed *PRND* gene polymorphisms in the Thoroughbred horse. However, we did not find polymorphisms in the *PRND* gene [30]. Because mating in the Thoroughbred horse is strictly regulated, it may cause nonpolymorphic genetic features of the *PRND* gene. Therefore, we investigated polymorphisms in the horse *PRND* gene in outbred breeds such as Jeju and Halla.

The Jeju horse is a Korean native horse and is registered and protected as Natural Monument No. 347. According to molecular genetic studies of Korean native Jeju horses, some of the ancestors of Jeju are believed to be of Mongolian origin [31], and some Jeju horses are estimated to have an independent maternal lineage. Halla horses are a crossbreed of the Jeju and Thoroughbred horses, which has been deemed a better breed of horses by the National Institute of Animal Science for horse racing. The lineage of Jeju horses is managed by the Jeju Horse Database System [32], operated by the Livestock Promotion Agency of Jeju Special Self-Governing Province. According to the Livestock Promotion Agency, 5223 Jeju horses were registered and are being raised in 613 breeding farms so far. In addition, 5201 Halla horses were registered and are being raised in 662 breeding farms. The habitat of Jeju and Halla horses is Jeju Island, which is a small island with an area of 1845 km^2^. Interestingly, we found a total of two novel SNPs, including one nonsynonymous SNP (c.331G > A, A111T) and one synonymous SNP (c.411G > C), in the ORF of the horse *PRND* gene. Unlike Thoroughbred horses, which are distributed in a wide range of habitats, SNPs were first discovered in Jeju and Halla horses despite their limited range of habitats. It can be assumed that the Jeju horse may have evolved longer than the Thoroughbred horse or maintained by free crossing.

According to a previous study, prion disease-susceptible species, such as sheep and goats, show strong LD between SNPs in the *PRNP* and *PRND* genes [27,28]. In contrast, prion disease-resistant species, such as dogs, show low LD values between these two genes. In this study, we first discovered a weak LD value between horse *PRNP* and *PRND* SNPs. A weak LD value may be regarded as a common feature of prion disease-resistant species (e.g., dogs and horses) [29]. However, the study is limited to a few breeds of only one alleged resistant species and should be extended to more breeds and/or other resistant species. Thus, careful validation is needed in future study.

In addition, prion disease-resistant species showed weak LD values despite the short physical map distance between the *PRNP* gene and the *PRND* gene (humans: 20 kb; sheep: 25 kb; goats: 25 kb; dogs: 17 kb; horses: 16 kb) (Figure 4). Interestingly, although there was a short physical map distance between the *PRNP* and *PRND* genes, weak LD between these two genes was identified in prion disease-resistant animals (Table 7). Strong genetic linkage between *PRNP* and *PRND* genes in prion disease-susceptible animals indicated that prion disease-susceptible animals were selected with prion disease-contributable alleles of *PRNP* and *PRND* genes. Thus, evaluation of susceptibility to prion disease according to alleles of polymorphisms in the *PRNP* and *PRND* genes will be highly needed in the future. 

## 5. Conclusions

In conclusion, we found a total of two novel SNPs, one nonsynonymous SNP (c.331G > A, A111T) and one synonymous SNP (c.411G > C), in the *PRND* gene in Jeju and Halla horses. The genotype and allele distributions of horse *PRND* SNPs were significantly different between Jeju, Halla, and Thoroughbred horses. In addition, we measured LD between SNPs in the *PRNP* and *PRND* genes and found a weak LD value. Furthermore, we investigated the physical map distance between the *PRNP* and *PRND* genes in prion disease-resistant and -susceptible species. Notably, although there was a short physical map distance between the *PRNP* and *PRND* genes, weak LD between these two genes was identified in prion disease-resistant animals. To the best of our knowledge, we provide evidence that a weak LD value could be a feature of prion disease-resistant animals.

## Figures and Tables

**Table 1:** Genotype and allele frequencies of the *PRND* c.331G > A polymorphism in Jeju, Halla, and Thoroughbred horses.

Breed	Total, n	Genotype Frequency, n (%)			*p*-Value	Allele Frequency, n (%)		*p*-Value	HWE	Ref
		GG	GA	AA		G	A			
Jeju	140	83 (59.3)	52 (37.1)	5 (3.6)	-	218 (77.9)	62 (22.1)	-	0.360	Current study
Halla	80	78 (97.5)	2 (2.5)	0 (0)	<0.0001	158 (98.8)	2 (1.2)	<0.0001	0.909	Current study
Thoroughbred	242	242 (100)	0 (0)	0 (0)	<0.0001	484 (100)	0 (0)	<0.0001	NA *	[30]

* NA, Not available.

**Table 2:** Genotype and allele frequencies of the *PRND* c.411G > C polymorphism in Jeju, Halla, and Thoroughbred horses.

Breed	Total, n	Genotype Frequency, n (%)			*p*-Value	Allele Frequency, n (%)		*p*-Value	HWE	Ref
		GG	GC	CC		G	C			
Jeju	140	130 (92.9)	10 (7.1)	0 (0)	-	270 (96.4)	10 (3.6)	-	0.661	Current study
Halla	80	79 (98.8)	1 (1.2)	0 (0)	0.06	159 (99.4)	1 (0.6)	0.0631	0.955	Current study
Thoroughbred	242	242 (100)	0 (0)	0 (0)	<0.0001	484 (100)	0 (0)	0.0022	NA	[30]

**Table 3:** Haplotype frequency of two *PRND* polymorphisms in Jeju and Halla horses.

Haplotype	Frequency		*p*-Value
	Jeju	Halla	
GG	208 (0.743)	157 (0.981)	
AG	62 (0.221)	2 (0.013)	<0.0001
GC	10 (0.036)	1 (0.006)	

**Table 4:** Prediction of the deleterious effect of the nonsynonymous polymorphism in the horse *PRND* gene by PolyPhen-2, PROVEAN, and PANTHER.

Variation	Method	Score	Prediction
c.331G > A (A111T)	PolyPhen-2	0.006	Benign
	PROVEAN	−0.969	Neutral
	PANTHER	176	Probably benign

**Table 5:** Linkage disequilibrium (LD) between two polymorphisms in the *PRND* gene in Jeju and Halla horses.

	Jeju		Halla		
	c.331G > A (Codon 111)	c.411G > C (Codon 137)	c.331G > A (Codon 111)	c.411G > C (Codon 137)	
c.331G > A (codon 111)	-	1.0	-	1.0	D’
c.411G > C (codon 137)	0.011	-	0	-	*r* ^2^

**Table 6:** Linkage disequilibrium (LD) between single nucleotide polymorphisms (SNPs) in the *PRNP* and *PRND* genes with *r*^2^ values in Jeju and Halla horses.

PRNP		PRND		
	Jeju		Halla	
	c.331G > A (Codon 111)	c.411G > C (Codon 137)	c.331G > A (Codon 111)	c.411G > C (Codon 137)
c.-3A > G	0.005	0.002	0.025	0.001
c.301T > A (codon 101)	0.0	0.0	0.0	0.0
c.525C > A (codon 175)	0.089	0.003	0.01	0.005
c.570G > A (codon 190)	0.002	0.002	0.003	0.002

**Table 7:** Genetic linkage between *PRNP* and *PRND* genes in several animals.

Common Name	Scientific Name	Prion Disease Susceptibility	Method	Genetic Linkage	References
Human	*Homo sapiens*	Susceptible animal	Pairwise disequilibrium	Strong linkage	[37]
Sheep	*Ovis aries*	Susceptible animal	Haplotype analysis	Strong linkage	[27]
Goat	*Capra hircus*	Susceptible animal	Pairwise disequilibrium	Strong linkage	[28]
Dog	*Canis lupus familiaris*	Resistant animal	Pairwise disequilibrium	Weak linkage	[29]
Horse	*Equs canbalus*	Resistant animal	Pairwise disequilibrium	Weak linkage	Current study

## References

[1] Prusiner S.B. (1998). Prions. Proc. Natl. Acad. Sci. USA.

[2] Prusiner S.B. (2006). The Priori Diseases. Brain Pathol..

[3] DeArmond S.J., Bajsarowicz K. (2010). PrPSc accumulation in neuronal plasma membranes links Notch-1 activation to dendritic degeneration in prion diseases. Mol. Neurodegener..

[4] Crozet C., Béranger F., Lehmann S. (2008). Cellular pathogenesis in prion diseases. Veter. Res..

[5] Kim Y.-C., Jeong B.-H. (2017). The first report of prion-related protein gene (PRNT) polymorphisms in goat. Acta Veter. Hung..

[6] Kim Y.-C., Jeong B.-H. (2018). First report of prion-related protein gene (PRNT) polymorphisms in cattle. Veter. Rec..

[7] Collee J.G., Bradley R. (1997). BSE: A decade on—Part I. Lancet.

[8] Lloyd S., Mead S., Collinge J. (2011). Genetics of Prion Disease. Chem. Diagn..

[9] Vaccari G., Panagiotidis C.H., Acin C., Peletto S., Barillet F., Acutis P.L., Bossers A., Langeveld J., Van Keulen L., Sklaviadis T. (2009). State-of-the-art review of goat TSE in the European Union, with special emphasis on PRNP genetics and epidemiology. Veter. Res..

[10] Kim Y.-C., Kim S.-K., Jeong B.-H. (2019). Scrapie susceptibility-associated indel polymorphism of shadow of prion protein gene (SPRN) in Korean native black goats. Sci. Rep..

[11] Kim Y.-C., Jeong B.-H. (2018). The first report of polymorphisms and genetic characteristics of the prion protein gene (PRNP) in horses. Prion.

[12] Jeong B.-H., Lee K.-H., Kim N.-H., Jin J.-K., Kim J.-I., Carp R.I., Kim Y.-S. (2005). Association of sporadic Creutzfeldt–Jakob disease with homozygous genotypes at PRNP codons 129 and 219 in the Korean population. Neurogenetics.

[13] Jeong B.-H., Kim Y.-S. (2014). Genetic Studies in Human Prion Diseases. J. Korean Med Sci..

[14] Belt P.B.G.M., Muileman I.H., Schreuder B.E.C., Ruijter J.B.-D., Gielkens A.L.J., Smits M. (1995). Identification of five allelic variants of the sheep PrP gene and their association with natural scrapie. J. Gen. Virol..

[15] Hunter N., Foster J.D., Goldmann W., Stear M.J., Hope J., Bostock C. (1996). Natural scrapie in a closed flock of Cheviot sheep occurs only in specific PrP genotypes. Arch. Virol..

[16] Ekateriniadou L.V., Panagiotidis C.H., Terzis A., Ploumi K., Triantafyllidis A., Deligiannidis P., Triantaphyllidis C., Sklaviadis T. (2007). Genotyping for PrP gene polymorphisms in rare Greek breeds of sheep. Veter. Rec..

[17] Goldmann W., Martin T., Foster J., Hughes S., Smith G., Hughes K., Dawson M., Hunter N. (1996). Novel polymorphisms in the caprine PrP gene: A codon 142 mutation associated with scrapie incubation period. J. Gen. Virol..

[18] Kim S.-K., Kim Y.-C., Won S.-Y., Jeong B.-H. (2019). Potential scrapie-associated polymorphisms of the prion protein gene (PRNP) in Korean native black goats. Sci. Rep..

[19] Baylis M., Goldmann W. (2004). The genetics of scrapie in sheep and goats. Curr. Mol. Med..

[20] Aguilar-Calvo P., Garcia C., Espinosa J.C., Andréoletti O., Torres J.M. (2015). Prion and prion-like diseases in animals. Virus Res..

[21] Fernández-Borges N., Eraña H., Castilla J. (2018). Behind the potential evolution towards prion resistant species. Prion.

[22] Vidal E., Fernández-Borges N., Eraña H., Parra B., Pintado B., Sánchez-Martín M.A., Charco J.M., Ordóñez M., Pérez-Castro M.A., Pumarola M. (2020). Dogs are resistant to prion infection, due to the presence of aspartic or glutamic acid at position 163 of their prion protein. FASEB J..

[23] Perez D.R., Damberger F.F., Wüthrich K. (2010). Horse Prion Protein NMR Structure and Comparisons with Related Variants of the Mouse Prion Protein. J. Mol. Boil..

[24] Sanchez-Garcia J., Fernandez-Funez P. (2018). D159 and S167 are protective residues in the prion protein from dog and horse, two prion-resistant animals. Neurobiol. Dis..

[25] Zhang J. (2011). The Structural Stability of Wild-type Horse Prion Protein. J. Biomol. Struct. Dyn..

[26] Sigurdson C.J., Joshi-Barr S., Bett C., Winson O., Manco G., Schwarz P., Rulicke T., Nilsson K.P., Margalith I., Raeber A. (2011). Spongiform encephalopathy in transgenic mice expressing a point mutation in the beta2-alpha2 loop of the prion protein. J. Neurosci..

[27] Mesquita P., Batista M., Marques M.D.R., Santos I.C., Pimenta J., Pereira M.S., Carolino I., Silva M.D.F.S., Sousa M.C.O., Gama L.T. (2010). Prion-like Doppel gene polymorphisms and scrapie susceptibility in portuguese sheep breeds. Anim. Genet..

[28] Jeong M.-J., Kim Y.-C., Jeong B.-H. (2018). Prion-like protein gene (PRND) polymorphisms associated with scrapie susceptibility in Korean native black goats. PLoS ONE.

[29] Won S.-Y., Kim Y.-C., Kim K., Kim A.-D., Jeong B.-H. (2019). The First Report of Polymorphisms and Genetic Features of the prion-like Protein Gene (PRND) in a Prion Disease-Resistant Animal, Dog. Int. J. Mol. Sci..

[30] Jeong M.-J., Jeong B.-H. (2019). No polymorphisms in the coding region of the prion-like protein gene in Thoroughbred racehorses. Acta Veter. Hung..

[31] Yang Y.H., Kim K.I., Cothran E.G., Flannery A.R. (2002). Genetic diversity of Cheju horses (Equus caballus) determined by using mitochondrial DNA D-loop polymorphism. Biochem. Genet..

[32] http://allhorse.kra.co.kr/ked/index.jsp.

[33] Kim Y.-C., Jeong B.-H. (2019). In Silico Evaluation of Acetylation Mimics in the 27 Lysine Residues of Human Tau Protein. Curr. Alzheimer Res..

[34] Kim Y.-C., Jeong M.-J., Jeong B.-H. (2019). Strong association of regulatory single nucleotide polymorphisms (SNPs) of the IFITM3 gene with influenza H1N1 2009 pandemic virus infection. Cell. Mol. Immunol..

[35] Adzhubei I., Jordan D.M., Sunyaev S.R. (2013). Predicting Functional Effect of Human Missense Mutations Using PolyPhen-2. Curr. Protoc. Hum. Genet..

[36] Kim Y.-C., Jeong M.-J., Jeong B.-H. (2018). The first report of genetic variations in the chicken prion protein gene. Prion.

[37] Mead S., Mahal S.P., Beck J., Campbell T., Farrall M., Fisher E., Collinge J. (2001). Sporadic—But Not Variant—Creutzfeldt-Jakob Disease Is Associated with Polymorphisms Upstream of PRNP Exon 1. Am. J. Hum. Genet..

